# Evaluation of teaching method for fluid mechanics course in engineering education

**DOI:** 10.1016/j.mex.2023.102380

**Published:** 2023-09-14

**Authors:** Yu Chen, Shaopeng Kang, Zhenhua Han, Kailei Liu, Hongchang Wang, Kai Wu

**Affiliations:** a School of Mechanical Engineering, Jiangsu University of Technology, Changzhou 213001, China; b School of Mechanical Engineering, Nanjing University of Science and Technology, Nanjing 210094, China

**Keywords:** Diversified teaching method, Evaluation, Fluid mechanics course, Engineering education, Diversified teaching method

## Abstract

The professional technology training of student is the important objective of engineering education, which could present the specialized ability in the future. This task is conducted to evaluation of teaching methods for fluid mechanics course in the mechanical engineering. With the teaching practice, the advantage of teaching method is found, and the teaching quality can be revealed by the scores of integrated test. The improvement of teaching quality is contribute the development of social. According to the self-assessment, the effects of teaching method on the professional course is revealed.

• The diversified teaching method is advantage to the understanding of theory and knowledge of application.
• The practical training is suitable to develop the inner potential and innovation of different students.
• The method allows the investigation of teaching method evaluation for the other courses with practicality.

## Graphical abstract

**Figure:**
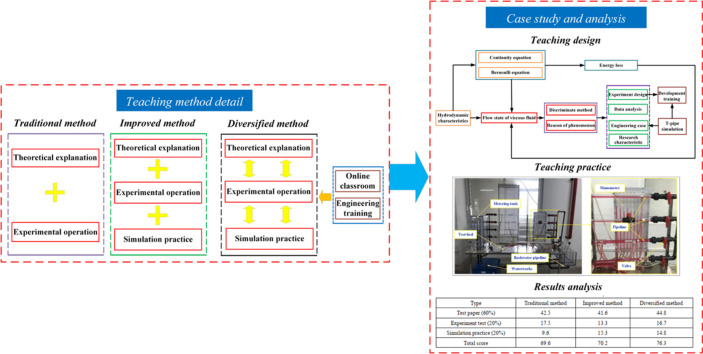

Specifications table

**Table:** 

Subject area:	Social science
More specific subject area:	Teaching method of professional course
Method name:	Diversified teaching method
Name and reference of original method:	N/A
Resource availability:	N/A

## Method details

### Background

The development of science and technology improves the requirement of professional person. As the foundation of skill training, the education of professional course plays an important role for the special technology [1], [2], [3]. The teaching method is close related with the teaching quality. For the professional course in the engineering education, the content of course includes the various parts, which can be explained by theoretical calculation, experiment operation and innovative design. The study of course content is just like the foundation of building, which is important for professional development of student in the future. Fluid mechanics is the professional basic course in the mechanical engineering and the content will count for many sequent courses. Then, it is important to find a suitable teaching method to conduct this course, which would be helpful for the study of relative course.

In recent years, the education researchers have attention for the traditional practical course with the advanced technology [4], [5], [6], [7], [8]. The simulation approach was introduced into the course of multibody dynamic by Ha [9]. The calculation cases of energy, impulse and momentum are conducted and the results showed that the students were attracted with this method and paid more attention for this course. Craifaleanu [10] extended the engineering mechanics course with the virtual laboratory applications, which would improve the ability of mechanical calculation and simulation. In addition, the comprehensive applied ability is the important role in the education of personnel training, which is concerned by researchers [11], [12], [13], [14]. The experiment investigation and CAD courses were introduced into the mechanical design course by Garikano [15]. The results showed that the trainees studied the design theory and conducted the virtual model at the same time, which listed the advantage of this method obviously. As the development of computer simulation systems, the related courses of computer (VR, multisource information fusion and 3D dynamic view) were compared with the fundamentals of physics course by Wang [16]. In this study, it was easily seen that the advanced technology of related courses solved the mathematical model quickly and the physical evolution of task could be described effectively, which provided a new perspective of practical education. However, there is not a teaching method that can satisfied the learning of engineering technology for the students. The traditional method cannot be close related with the modern learning approach of students.

The objective of this work is to develop the evaluation approach for evaluating and searching the suitable teaching method in the fluid mechanism course, which provides a new way for the professional technology course. The results represent the advantage of diversified teaching method, which is suitable to the development of interdisciplinary integration. In the basic course of mechanical engineering, the diversified teaching method could provide a better teaching quality for study of professional technology, which can be extended to the similar courses.

### Method

**Fig. 1:**
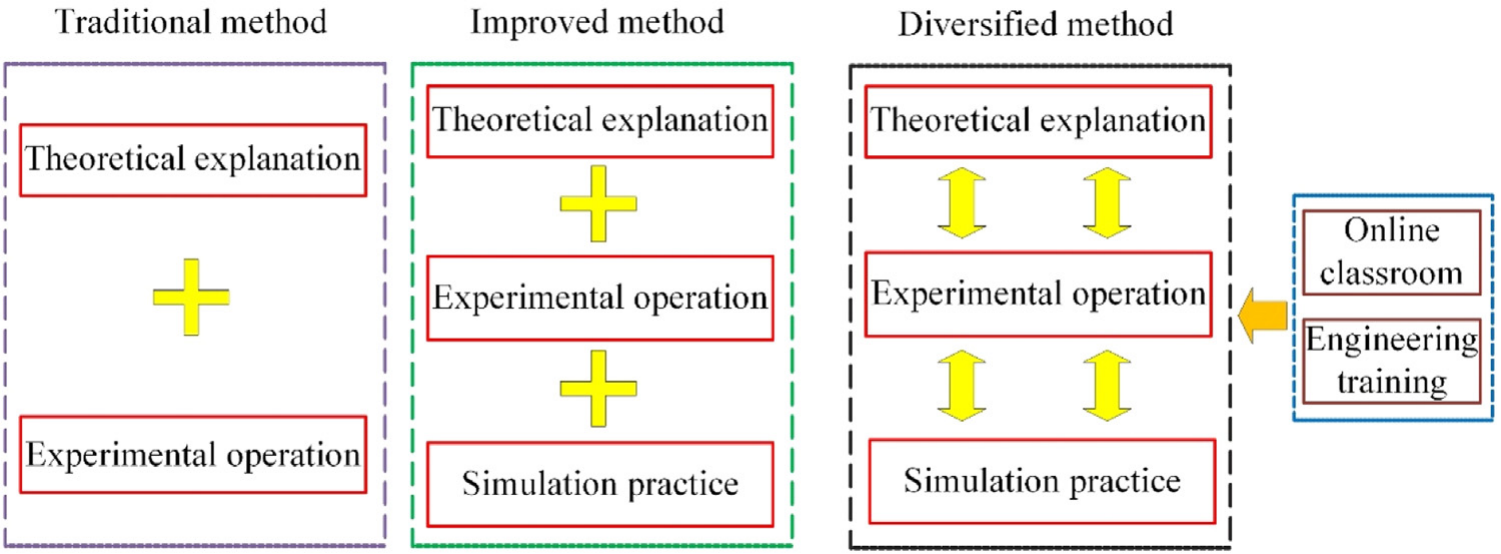
Comparison of different teaching methods.

For the fluid mechanics course, the teaching method includes traditional method, improved method and diversified method, as shown in Fig. 1. The traditional teaching method consists of theoretical explanation and experimental operation. The study of theoretical explanation and experimental operation is separated in the teaching process, and the experimental operation is always defined after the theoretical explanation. The teaching step cannot be tight by the time interval, and some contents cannot be conducted by experiment operation. In order to obtain a better teaching results, the simulation practice is introduced into the traditional teaching method, which is named improved method. As well know, it is advantage to understand the content of fluid mechanics by the observation of experiment phenomenon. The simulation practice can provide the virtual phenomenon by the simulation software, which is suitable to conduct the case without experiment operation. As the investigation of teaching method for fluid mechanics course, the personal ability of students is different, and the proportion time of simulation practice brings the trouble in the process of teaching. Then, the diversified teaching method is proposed with the online classroom and engineering training. The teaching content of fluid mechanics course is conducted by the fixed time and free time. And then, the engineering training provides the helpful for the understanding and application of knowledge. The introduction of engineering case study is established the connection between basic theory of this course and engineering application, which is adapted to the requirement of innovative and applied talents. Moreover, students can manage the learning time and process by themselves, and the boring environment of learning would be improved. There is a saying that interest is the best teacher. Some students will take more time for improving the practice skill. In addition, the different teaching methods is employed in the fluid mechanics course and the scores of students is evaluated by the integrated test, which includes theory, practice and innovative ability.

## Task procedure

### Case study

**Fig. 2:**
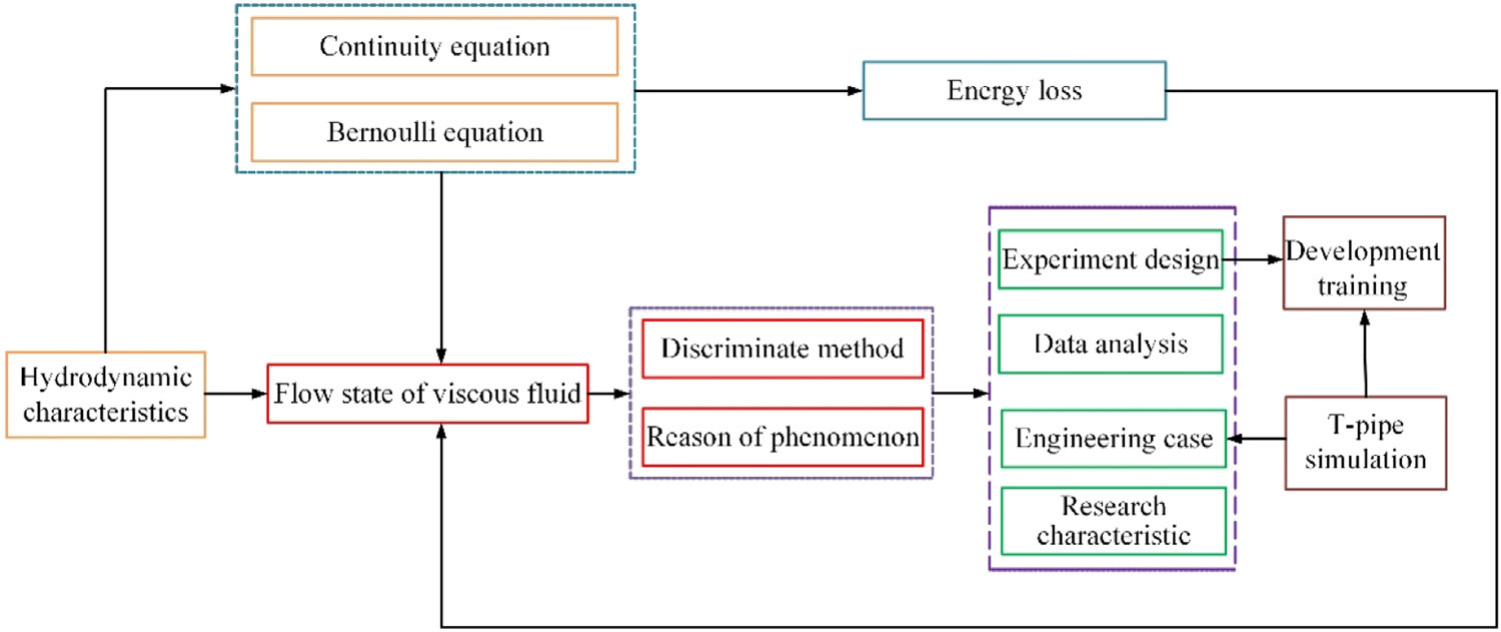
Teaching design of case study.

Before the start of teaching practice, the teacher is to be known about the content of fluid mechanics course (Flow state of viscous fluid). During the preparation of course, the students could read the new knowledge by themselves and the teaching design of this course is plotted in Fig. 2. In order to observe the different flow states of viscous fluid, the experiment section is introduced into the teaching.

• Traditional method: The first step of traditional method is the theoretical explanation, which describes the characteristics of viscous fluid and the discriminate method of different states. Then, the experiment operation can promote the understanding of flow states in the next step.
• Improved method: Compared with the traditional method, the simulation case is introduced into the class and the flow state of viscous fluid in the tube is conducted by CFD technology. The students can see the characteristics of viscous fluid in the computer and the effects of structural parameters and operation condition on the flow state of viscous fluid are also performed.
• Diversified method: Based on the above methods, the development training is added in the teaching of flow state of viscous fluid. Meanwhile, the learning resources is uploaded into the network learning platform before the class. The students study the simulation software in the space time, and the development training is conducted after class.

(1) Explanation of basic theory

With the characteristics of viscosity in the actual fluid, the impact force of fluid molecules would appear in the flow path, which is different to the feature of ideal fluid. The appearance of impact force in the flow path could cause the energy loss in the process of flow. As the variation of viscosity, density and velocity, the flow state of viscous fluid has different characteristics, which are laminar flow and turbulence flow. With the state of laminar flow, the streamline of fluid is similar to straight line. As the increase of velocity, the streamline has the vibration feature and the turbulence state appears. In order to describe the flow state of fluid accurately, the Reynolds number is proposed to confirm the flow state of fluid in the pipeline, which is written as:

(1) $$R_{e}=\frac{vd}{\upsilon }$$

where *v* is the average velocity of fluid, *d* denotes the diameter of pipeline, and υ is the viscosity.

(2) Experiment setup

**Fig. 3:**
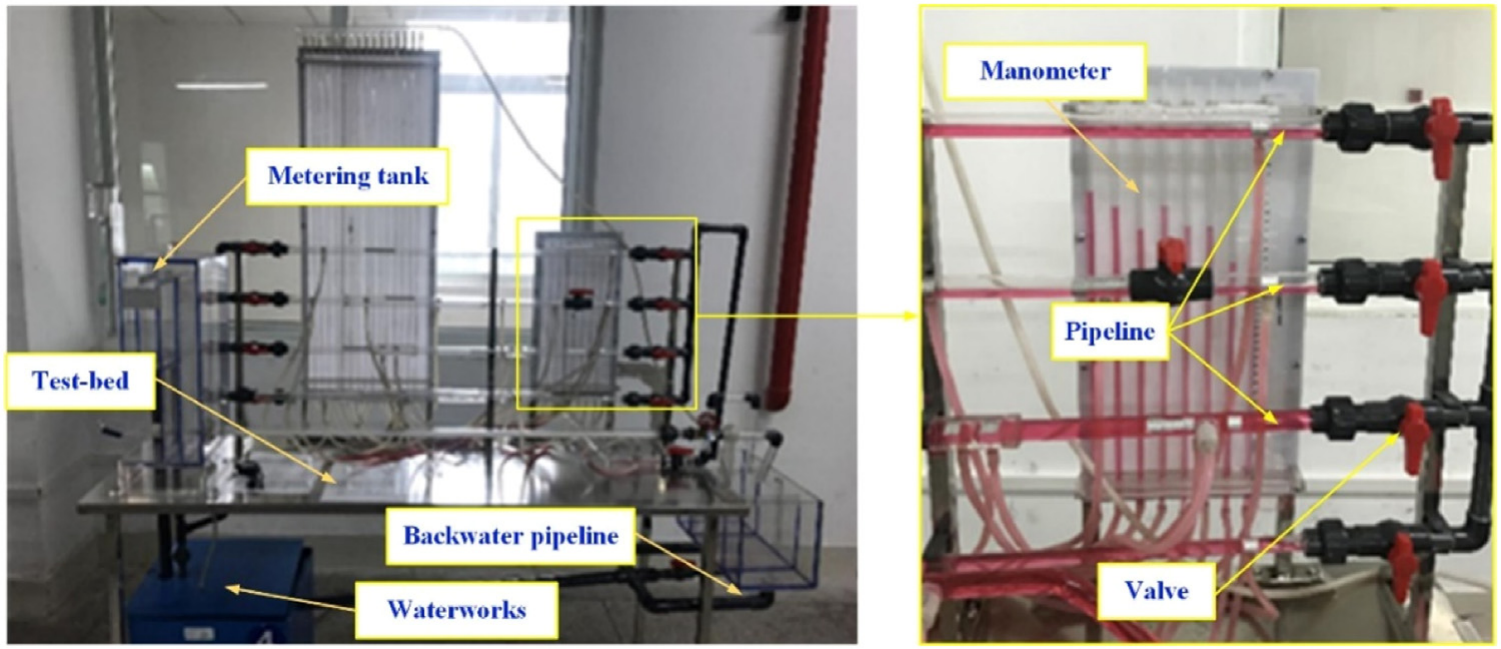
Model of resistance in the flow path.

**Fig. 4:**
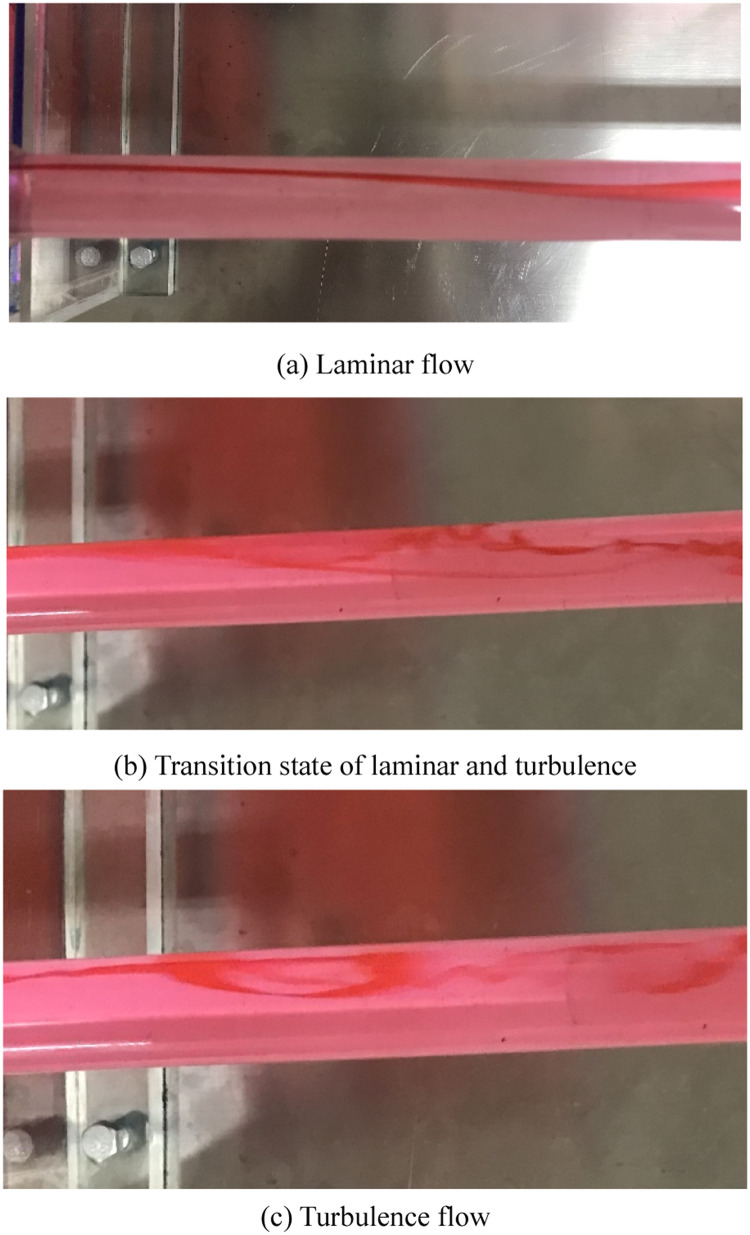
Flow state of fluid.

The experiment of flow state for fluid would reveal the relationship between Reynolds number and streamline. Then, the experiment platform is established, as shown in Fig. 3. The devices of experiment are installed the test-bed and the waterworks is located under test-bed, which could provide the circulating water for the test. The metering tank could record the liquid level height and manometer displays the pressure of fluid. The valve would control the velocity of fluid in the pipeline. Moreover, the red liquid is easy to the collection of test data. Then, the effects of flow velocity on the frictional head coefficient of fluid are conducted. Meanwhile, the marked fluid is employed to recognize the flow state of fluid and it is decided by the flow velocity. The flow states of fluid are displayed in Fig. 4. The experiment results illustrate that the pressure of fluid is sensitive to the flow state and the differential pressure appears in the manometer. Based on the test data, the characteristics of flow state are obtained in the different velocities. During the study of fluid mechanics course, students understand the definition of discriminate method for flow state and recognize the relationship of flow velocity, flow state and Reynolds number deeply.

(3) Simulation and development training of engineering case

**Fig. 5:**
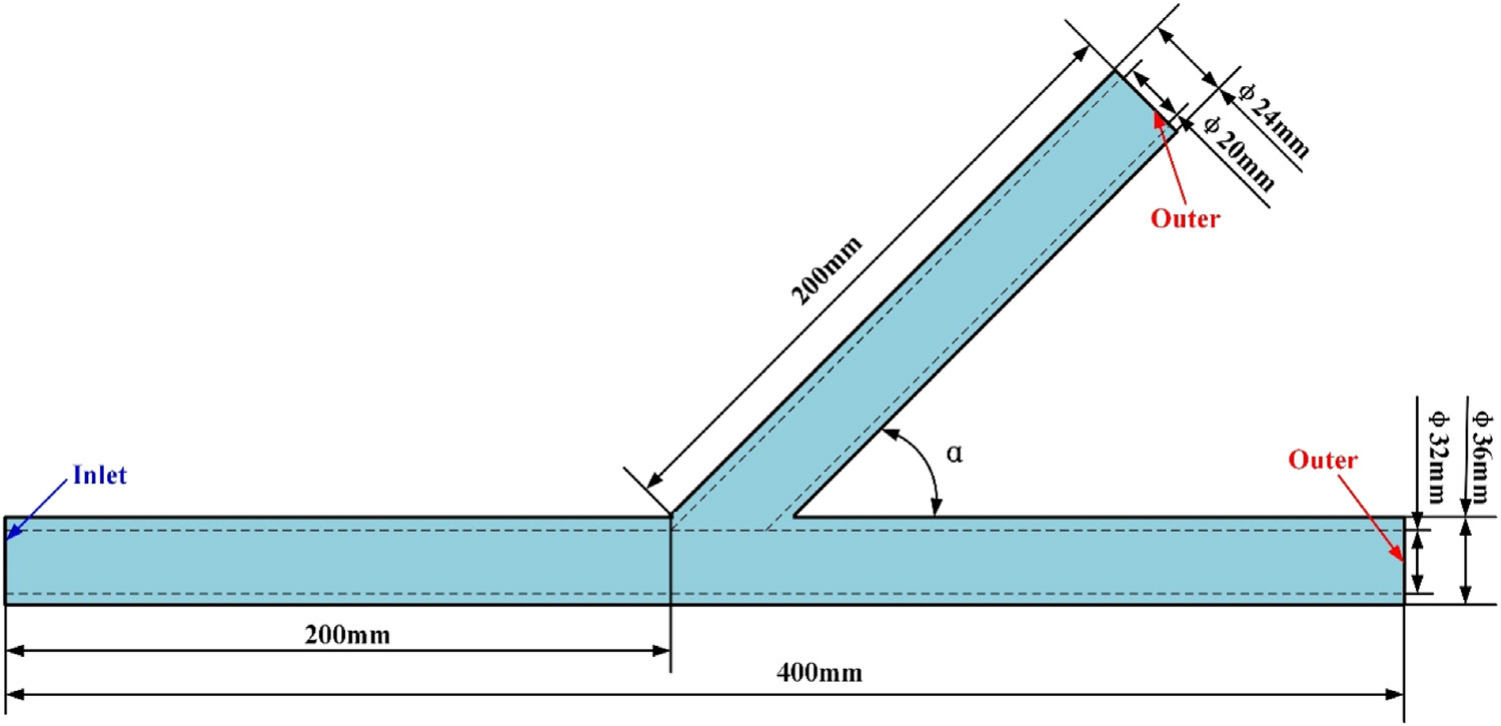
Simulation model.

In order to enhance the application ability of students for hydrodynamic characteristics in the different flow state, the engineering case of fluid mechanics is developed. T-pipe is used to investigate the effects of geometry parameters on the hydrodynamic behavior of fluid, as shown in Fig. 5 and the parameters are given in Table 1.

**Table 1:** Simulation parameters.

Description	values	Description	values
Inlet velocity *v* (mm/s)	50	Angle α (°)	30∽90
Density of water *ρ*_W_ (kg/m^3^)	1000	Density of lubrication oil *ρ*_L_ (kg/m^3^)	890
Viscosity of water $\nu_{W}$ (Pa·s)	1.003 × 10^−6^	Viscosity of lubrication oil $\nu_{L}$ (Pa·s)	0.06

As the usual software of hydrodynamic simulation, Solidworks and Fluent are employed to the teaching of fluid mechanics course. Compared with the 2D model, the 3D model can provide a better visual effect for learning of student, and Solidworks can provide the service for the modeling with the requirement of engineering case. Then, the model is divided to many smaller elements and transferred into the Fluent software. According to the requirement of design, the boundary condition of calculation is defined and the solution of simulation is conducted.

**Fig. 6:**
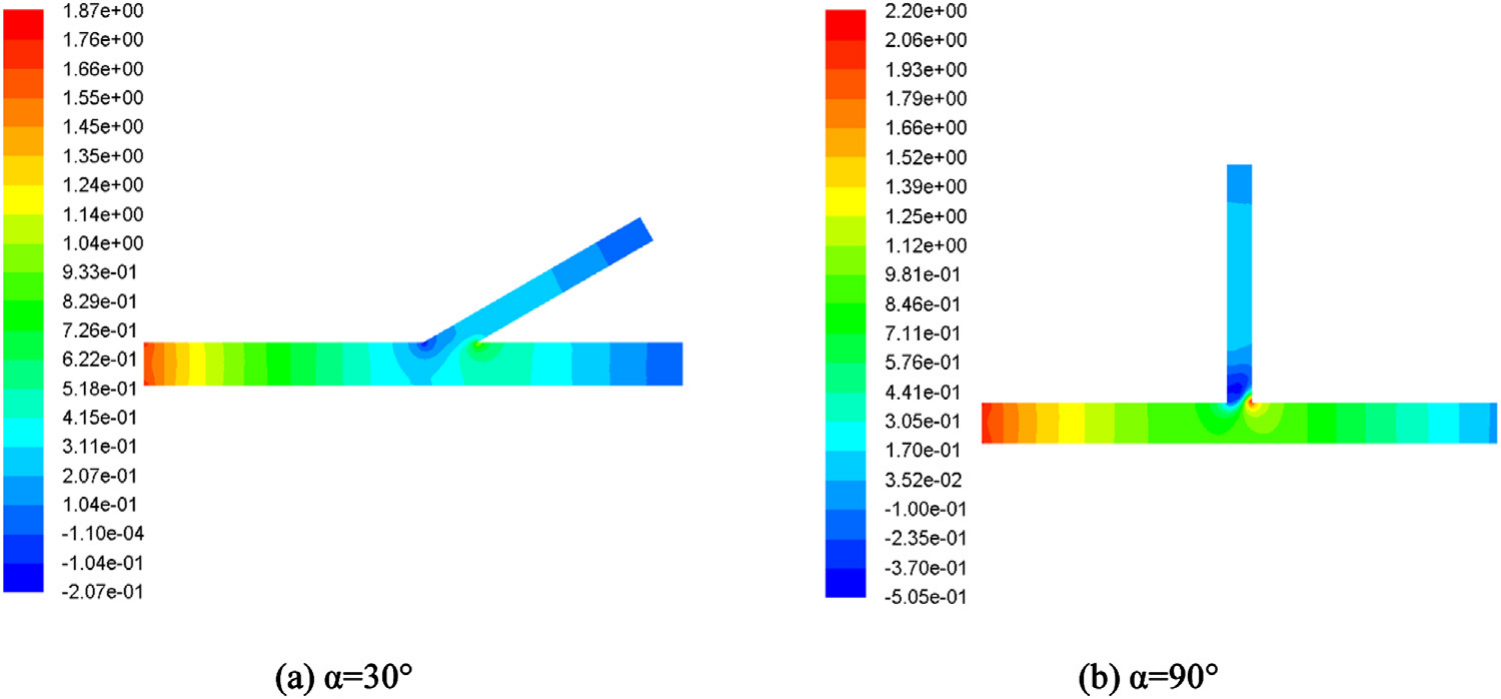
Simulation results of T-pipe.

Then, the simulation results is shown in Fig. 6. It is easy found that the angle has the obvious effects on the pressure distribution of water in the T-pipe. Although the maximum value of pressure is increased of the model (90°), the pressure gradient changes obviously at the same time. As well known, the pressure is close related with the flow velocity of fluid. Then, the velocity gradient of water has the characteristics of irregular various. Based on the Eq. (1), the flow state of fluid can be decided with the flow velocity and the turbulence flow should be appeared in the T-pipe.

### Results and analysis

Furthermore, the evaluation method is the key element for teaching method. A suitable evaluation method could provide the learning situation of students for teacher and remind the defect of study for students. In the fluid mechanics course, the multi-modes evaluation model is established, which is named “*N* + 1″. In the “*N* + 1″model, “1″ represents the test paper, which includes the essay questions of theory, explanation of fluid mechanics phenomenon and engineering calculation. “N” denotes the comprehensive evaluation system of course content, which includes classroom test, take-home test, practical training, development training and experiment test. This method records the learning process of students in the fluid mechanics course and evaluates the ability of students completely, which exploits the potential of students. Meanwhile, the students must build “Growth record card” by themselves. Then, the attention of study is conducted by students in the whole learning process and “Growth record card” could help students to review the knowledge of fluid mechanics course. The “Growth record card” consists of basic theory, example of calculation, design and building of experiment, and the outward development training. Based on the management of fluid mechanics course, the different types of scoring are added during the learning process. The evaluations of oneself and others are also introduced into the evaluation model, which would help students find the disadvantage of oneself and advantage of others. The ability test is conducted after class, and the scores of integrated test includes the theoretical calculation, design and operation of experiment, and research analysis of engineering case.

The results are list in Table 2 and the self-assessment of students is also conducted. According to the results, the total score of traditional method is similar to that of improved method. However, the experiment test score of traditional method is higher. The reason of this phenomenon is that the practice time is longer than other methods, and the optional skill of students is well. Due to the introduction of simulation section in the class, the time of experiment operation is decreased and the scores is lower. Moreover, the ability of simulation practice is worse for the traditional method. The students cannot obtain a better simulation skill and engineering application solely by themselves. The suitable explanation could provide helpful for the learning and the application of diversified method demonstrates the results. Compared with the results of different teaching methods, the scores and ability of engineering analysis for diversified method is more superior than other teaching methods. In addition, the questionnaire investigation of this course shows the viewpoint of students. The students find that the simulation practice and development training are the interesting section in the learning. And, the online classroom could provide more free time for learning and investigation, which is advantage to the consolidation and understanding of knowledge. Compared with the monotonous of traditional method, the diversified method is easier to attract attention of students. Then, the learning efficiency is improved and the connection between theory and practice is strengthened.

**Table 2:** Evaluation form of case study.

Type	Traditional method	Improved method	Diversified method
Test paper (60 %)	42.5	41.6	44.8
Experiment test (20 %)	17.5	13.3	16.7
Simulation practice (20 %)	9.6	15.3	14.8
Total score	69.6	70.2	76.3

### Recommendations

Teaching method is crucial to the professional technology in the study. The diversified method described in this paper can effectively improve the activeness motivation of students. With a case study, this method makes it possible to successfully perform the thinking and active of student in the fluid mechanics course. Moreover, the establishment of online classroom is advantage to the use of learning time for students. The interesting and freedom can provide helpful for learning efficiency of student. Then, the development practice of engineering case provide the helpful for the improvement of professional skill application in the future, which could maximize the advantage of technology. Moreover, the professional technology course of mechanical engineering has the similar way, which is also serviced to the engineering practice and training of professional person. The teaching method of fluid mechanics course could provide reference for the course construction of other courses.

## Declaration of Competing Interest

The authors declare that they have no known competing financial interests or personal relationships that could have appeared to influence the work reported in this paper.

## Data Availability

No data was used for the research described in the article.
